# Allosteric inhibition of HSP70 in collaboration with STUB1 augments enzalutamide efficacy in antiandrogen resistant prostate tumor and patient-derived models

**DOI:** 10.1016/j.phrs.2023.106692

**Published:** 2023-02-10

**Authors:** Pengfei Xu, Joy C. Yang, Shu Ning, Bo Chen, Christopher Nip, Qiang Wei, Liangren Liu, Oleta T. Johnson, Allen C. Gao, Jason E. Gestwicki, Christopher P. Evans, Chengfei Liu

**Affiliations:** a Department of Urologic Surgery, University of California, Davis, CA, USA; b Department of Urology, West China Hospital, Sichuan University, Sichuan, China; c Department of Pharmaceutical Chemistry, University of California, San Francisco, CA, USA; d University of California, Davis Comprehensive Cancer Center, CA, USA

**Keywords:** Prostate cancer, Enzalutamide resistance, HSP70, STUB1, Androgen receptor variant, Patient-derived model

## Abstract

Ubiquitin proteasome activity is suppressed in enzalutamide resistant prostate cancer cells, and the heat shock protein 70/STIP1 homology and U-box-containing protein 1 (HSP70/STUB1) machinery are involved in androgen receptor (AR) and AR variant protein stabilization. Targeting HSP70 could be a viable strategy to overcome resistance to androgen receptor signaling inhibitor (ARSI) in advanced prostate cancer. Here, we showed that a novel HSP70 allosteric inhibitor, JG98, significantly suppressed drug-resistant C4–2B MDVR and CWR22Rv1 cell growth, and enhanced enzalutamide treatment. JG98 also suppressed cell growth in conditional reprogramed cell cultures (CRCs) and organoids derived from advanced prostate cancer patient samples. Mechanistically, JG98 degraded AR/AR-V7 expression in resistant cells and promoted STUB1 nuclear translocation to bind AR-V7. Knockdown of the E3 ligase STUB1 significantly diminished the anticancer effects and partially restored AR-V7 inhibitory effects of JG98. JG231, a more potent analog developed from JG98, effectively suppressed the growth of the drug-resistant prostate cancer cells, CRCs, and organoids. Notably, the combination of JG231 and enzalutamide synergistically inhibited AR/AR-V7 expression and suppressed CWR22Rv1 xenograft tumor growth. Inhibition of HSP70 using novel small-molecule inhibitors coordinates with STUB1 to regulate AR/AR-V7 protein stabilization and ARSI resistance.

## 1. Introduction

Prostate cancer accounts for the highest number of new cancer diagnoses in men in the United States [1]. Although androgen deprivation therapy (ADT) serves as gold standard treatment for advanced prostate cancer, most cases eventually progress to castration-resistant prostate cancer (CRPC) [2]. In recent years, androgen receptor signaling inhibitor (ARSI), including abiraterone, enzalutamide, apalutamide, and darolutamide, have been used to treat CRPC. However, drug resistance is inevitable [3]. Many studies have shown that androgen receptor (AR) signaling pathways play an important role in driving CRPC progression and drug resistance through the aberrant amplification and/or overexpression of AR, AR mutations, and AR variants (AR-Vs) [4-6]. Importantly, emerging evidence indicates that AR-V7 plays a key role in promoting CRPC progression and inducing resistance to ARSI [7,8]. This AR variant is constitutively active and insensitive to antiandrogen treatment, conferring a growth advantage to CRPC in androgen-depleted environments and resulting in castration-resistant growth *in vivo* [9,10]. AR-V7 expression in CRPC often leads to resistance to standard endocrine therapy and is associated with poor prognosis, shorter progression-free survival (PFS), and decreased overall survival (OS) rates [11]. The correlation between AR-V7 and CRPC development has been well documented, making the modulation of AR-V7 expression and stability a potential and promising therapeutic target. However, AR-V7 specific inhibitors are currently unavailable.

AR-V7 structurally retains the N-terminal domain (NTD) and DNA-binding domain (DBD) but lacks the C-terminal ligand-binding domain (LBD). The C-terminal LBD is targeted by traditional endocrine therapy, which explains why enzalutamide is unable to exert its effects in AR-V7 positive tumors, consequently resulting in drug resistance [12]. AR-V7 is a client protein of the heat shock proteins (HSP40 and HSP70) [13], and its expression is regulated by post-translational modifications [14]. AR-V7 protein stability relative to ARSI resistance is regulated by the ubiquitin-proteasome system, and its homeostasis is sustained by the HSP70/STUB1 complex. HSP70 inhibition markedly disrupted AR and AR-V7 gene programs and re-sensitized resistant cells to enzalutamide and abiraterone treatment *in vitro* and *in vivo* [15]. In other preclinical studies, knockdown of HSP70 using siRNA induced massive cell death in breast cancer cell lines without toxicity to normal breast cells, suggesting that targeting HSP70 selectively induces tumor cell death [16]. Moreover, inhibition of GRP78, a HSP70 family member, causes endoplasmic reticulum (ER) stress and triggers the unfolded protein response (UPR), leading to cancer cell apoptosis and tumor growth retardation [17]. Therefore, the development of HSP70 inhibitors is a promising strategy for the treatment of CRPC.

To date, considerable effort has been devoted to the development of HSP70 inhibitors. Although drugs targeting the nucleotide-binding domain (NBD) and substrate-binding domain (SBD) of HSP70 have been developed, only the NBD inhibitor, rhodamine cyanine MKT-077, has been tested in cancer patient. This drug was shown to delay tumor growth in DU145 xenograft models [18], but phase I clinical trials were terminated because of severe nephrotoxicity [19,20]. The HSP70 allosteric inhibitors, JG98 and JG231, were derived from MKT-077 and inhibited HSP70 by preventing nucleotide exchange. This inhibition stabilizes the high-affinity HSP70/client interaction bound to adenosine diphosphate (ADP) and prevents client release from HSP70, promoting its eventual degradation by the proteasome system [21-24]. However, the effects of these drugs in combination with ARSI treatment for CRPC have not been fully investigated. In the present study, we examined the effects of these novel HSP70 inhibitors in combination with ARSI treatment and inhibition of AR/AR-V7 signaling using drug-resistant CRPC cell line, conditional reprogrammed cell cultures (CRCs), organoids, and xenograft tumor models. Our preclinical findings identified novel HSP70 allosteric inhibitors as potential treatment options for ARSI-resistant CRPC.

## 2. Materials and methods

### 2.1. Reagents and cell culture

C4–2B MDVR (C4–2B enzalutamide resistant) and CWR22Rv1 cells were maintained in RPMI1640, whereas HEK293 and IMR90 fibroblast cells were cultured in DMEM supplemented with 10% fetal bovine serum (FBS), 100 units/ml penicillin, and 0.1 mg/ml streptomycin. RWPE-1 cells were grown in Karotinocyte SFM medium (Gibco). All cell line experiments were performed within six months of receipt from the ATCC or resuscitation after cryopreservation. C4–2B cells were kindly provided and authenticated by Dr. Leland Chung Lab at Cedars-Sinai Medical Center (Los Angeles, CA, USA). C4–2B MDVR cells were maintained in medium containing 20 μM enzalutamide. Parental C4–2B cells were passaged alongside the resistant cells as an appropriate control [25,26]. All cell lines were routinely tested as mycoplasma-free by PCR and authenticated using the short tandem repeat (STR) method. All cells were maintained at 37 °C in a humidified incubator with 5% carbon dioxide. Enzalutamide, apalutamide, darolutamide, and abiraterone acetate were purchased from Selleck Chemical. JG98 and JG231 were synthesized as described and their identities confirmed by ^1^H NMR and LC-MS/MS. Purity was > 95%, as determined by HPLC [27].

### 2.2. Plasmids and cell transfection

For small interfering RNA (siRNA) transfection, cells were seeded at a density of 0.5 × 10^5 cells per well in 12-well plates or 2 × 10^5^ cells per well in 6-well plates and transfected with 20 nM siRNA (Invitrogen) targeting the STUB1 sequence (Catalog# 215046) or control siRNA (Catalog# 12935300) using Lipofectamine-iMAX (Invitrogen). The effect of siRNA-mediated gene silencing was examined using qRT-PCR and western blotting 2–3 days after transfection. Cells were transiently transfected with plasmids expressing AR-FL [28], AR-V7 [28], Flag-STUB1 (Sino Biological, Catalog# HG12496-NF), or HSP70 (HSPA1B, OriGene, Catalog# SC116767), using Lipofectamine 2000 (Invitrogen).

### 2.3. Western blot analysis

Whole cell protein extracts were resolved on SDS-PAGE, and proteins were transferred to nitrocellulose membranes. After blocking for 1 h at room temperature in 5% milk in PBS/0.1% Tween-20, membranes were incubated overnight at 4 °C with the following primary antibodies: AR (441, sc-7305, 1:1000 dilution, Santa Cruz Biotechnology, Santa Cruz, CA); ubiquitin (P4D1 and FL76, 1:1000 dilution, Santa Cruz Biotechnology, Santa Cruz, CA); STUB1 (C3B6, 1;1000 for WB, 1:100 for immunoprecipitation, Cell Signaling Technology Cat# 2080, RRID: AB_2198052); AR-V7 (AG10008, 1:1000, (Precision antibody Cat# AG10008, RRID:AB_2631057); FLAG^®^ M2 monoclonal antibody (F1804, 1:1000 for WB, 1:200 for IP, (Sigma-Aldrich Cat# F1804, RRID: AB 262044); c-Myc (18583, 1:1000, Cell Signaling Technology); HSP70 (4873, 1:1000, Cell Signaling Technology); tubulin (T5168, 1:5000, Sigma-Aldrich); actin (4970, 1:1000, Cell Signaling Technology); GAPDH antibody (2118, 1:1000, Cell Signaling Technology Cat# 2118, RRID: AB_561053). Tubulin, actin, and GAPDH were used as loading controls. Following incubation with secondary antibodies (W401 and W402, 1: 5000 dilution, Promega Cat# W4021, RRID: AB_430834), immunoreactive proteins were visualized using an enhanced chemiluminescence detection system (Millipore, Billerica, MA, USA).

### 2.4. Co-immunoprecipitation assay

Equal amounts of cell lysates (1500 μg) were immunoprecipitated overnight using 1 μg of AR-V7, AR (441) or FLAG M2 antibody with 50 μL of protein A/G agarose with constant rotation. The immunoprecipitants were washed twice with 1 ml 10 mM HEPES (pH 7.9), 1 mM EDTA, 150 mM NaCl, and 1% Nonidet P-40. The precipitated proteins were eluted with 30 μL of SDS-PAGE sample buffer by boiling for 10 min. The eluted proteins were electrophoresed on an 8% SDS-PAGE gel, transferred to nitrocellulose membranes, and probed with the indicated antibodies.

### 2.5. Dual immunofluorescence assay

1 × 10^4^ HEK293 cells were plated in 4-well Nunc^™^ Lab-Tek^™^ II Chamber Slides, transfected with AR-V7, HSP70, and Flag-STUB1 for 3 days, and then treated with JG98 with 5 μM MG132 for 16 h. The cells were fixed with 4% paraformaldehyde, permeabilized with 0.5% Triton X-100, and incubated with 1% bovine serum albumin (BSA) to block nonspecific binding. The slides were washed multiple times using phosphate-buffered saline with Tween 20 (PBST). Cells were incubated with anti-AR (N20, Santa Cruz Biotechnology) and anti-FLAG antibodies (Sigma) overnight. Intracellular AR-V7 was visualized using FITC-conjugated secondary antibodies, Flag-STUB1 was visualized using Texas red-conjugated secondary antibodies, and nuclei were visualized with DAPI using an all-in-one fluorescence microscope (BZ-X700).

### 2.6. Real-time quantitative RT-PCR

Total RNA was extracted using the RNeasy Mini Kit (Qiagen, Hilden, Germany). cDNA was prepared after digestion with RNase-free RQ1 DNase (Promega) and subjected to real-time reverse transcription-PCR (RT-PCR) using SsoFast Eva Green Supermix (Bio-Rad) according to the manufacturer’s instructions, as described previously [29]. Each reaction was normalized to the co-amplification of actin. Triplicate samples were run using the default settings of the Bio-Rad CFX-96 real-time cycler. The primer sequences are in Table S1.

### 2.7. Luciferase assay

C4–2B cells were transfected with pGL3-PSA6.0-Luc reporters, pRL-TK (TK promoter-Renilla luciferase construct as an internal control), or different constructs (AR-V7 and HSP70). The cells were stimulated with 1 mM DHT or treated with 2.5 and 5 μM JG98 or enzalutamide in charcoal stripped FBS. Cell lysates were subjected to luciferase assays using the Luciferase Assay System (Promega) as described previously [30].

### 2.8. Cell growth assay

CWR22Rv1, C4–2B MDVR, IMR90, and RWPE-1 cells were seeded in 12-well plates at a density of 0.2 × 10^5^ cells/well in RPMI 1640 media containing 10% FBS and treated with various concentrations of JG98. The total number of cells was counted to calculate the percentage of cell survival. CWR22Rv1 and C4–2B MDVR cells were treated with JG98, enzalutamide, or a combination of both for 3 or 5 days, and the total cell numbers were counted. Cell viability for the UCD1172, UCD1172CR, UCD1173, UCD1177, and UCD243009 cells after JG98 and enzalutamide treatment was determined with the cell counting kit-8 (CCK-8) system.

### 2.9. Clonogenic assay

CWR22Rv1, C4–2B MDVR, and UCD1172CR cells were plated at an equal density (400 cells/dish for CWR22Rv1 and C4–2B MDVR cells and 5000 cells for UCD1172CR cells) in 6-well plates treated with different dose of JG98 with or without 20 μM enzalutamide for 3 weeks, and the medium was changed every 7 days. The colonies were rinsed with PBS before staining with 0.5% crystal violet/4% formaldehyde for 30 min and the number of colonies was counted.

### 2.10. RNA-seq data analysis

RNA was extracted from C4–2B MDVR cells treated with 2.5 and 5 μM JG98 for 24 h. RNA-seq libraries from 1 μg of total RNA were prepared using the Illumina Tru-Seq RNA Sample according to the manufacturer’s instructions. The mRNA-Seq paired-end library was prepared using Illumina NGS on a HiSeq 4000:2 × 150 cycles/bases (150 bp, PE). Around 30 M of reads/sample were obtained. Data analysis was performed using a Top Hat-Cufflinks pipeline and sequence read mapping/alignment was performed using HISAT. StringTie Data were mapped and quantified for 27,044 unique genes/transcripts. Gene and transcript expression was quantified as FPKM (Fragments Per Kilobase of transcript per million mapped reads). Principal Component Analysis (PCA) was conducted on the FPKM gene-level data for all genes/transcripts that passed the filter (Filtered on Expression > 1, ∣ log2 ((FKPM_1_ +0.1)/(DMSO+0.1),2) ∣ > 0.25, and kept 0→values and values→0) in the Raw Data. The genes commonly regulated by JG98 treatments were clustered with the Hierarchical Clustering algorithm using the R.

### 2.11. Gene set enrichment analysis (GSEA)

GSEA (SeqGSEA, RRID:SCR_005724) was performed using Java desktop software (http://software.broadinstitute.org/gsea/index.jsp) as described previously (29). Genes were ranked according to the shrunken limma log2 fold change, and the GSEA tool was used in the ’pre-ranked’ mode with all default parameters. The KEGG-ubiquitin-mediated proteolytic pathway was used for GSEA analysis.

### 2.12. Patient-derived xenografts (PDXs), conditional reprogramed cell cultures (CRCs), and organoid cultures

All human sample collection has been complied with all relevant ethical regulations for work with human participants at UC Davis. The Institutional Review Board (IRB) approved protocol (protocol number is GU-001) covered the patient specimen acquisition. All the patient provided permission to access residual tissue through the consent process. Experimental procedures involving animals were approved by the Institutional Animal Care and Use Committee of UC Davis complied with ARRIVE guidelines and ethical regulations and humane endpoints (animal protocol number is #19796). 5-week-old male NOD.Cg-Prkdcscid Il2rgtm1Wjl/SzJ (NSG, Envigo) mice was inoculated with tumor specimens from patients to establish PDX. Renal capsule and/or the prostate were the implantation sites. Once the xenografts were established, tumors were propagated in 5-week-old male NSG or C.B-17/lcrHsd-PrkdcscidLystbg-J (SCID, Envigo) mice to further generate CRCs. Primary cells from malignant human prostate tissue or PDX tumors were isolated according to a previously described protocol [31]. Briefly, tissue was minced and digested with collagenase/hyaluronidase/dispase at 37 °C for 1–3 h. The dissociated cell suspension was filtered through a 100 μm cell strainer and collected. Cells were plated in a mixture of complete F-medium/conditioned medium from irradiated J2 cultures supplemented with 10 μM Y-27632. Subculturing was performed with trypsin treatment when required.

For organoid cultures, PDX tumor tissues were collected and cut into 2–4 mm^3^. Tumors were digested using collagenase IV (STEMCELL) and incubated at 37 °C for 30 min until tumor cells were dispersed. Advanced DMEM (ADMEM) medium supplemented with 1 × GlutaMAX (Gibco), 1 M HEPES (Gibco), 100 u/ml penicillin, and 0.1 mg/ml streptomycin was added to the cell suspension and then filtered through 40 μm cell strainers to obtain a single-cell suspension. The cells were then centrifuged and resuspended in ADMEM complete medium containing GlutaMAX (Gibco), 100units/ml penicillin, 0.1 mg/ml streptomycin, B27 (Gibco), N-Acetylcysteine (Thermo Scientific), Human Recombinant EGF (Thermo Scientific), Recombinant FGF-10 (Invitrogen), A-83–01 (Tocris), SB202190 (Bioscience), Nicotinamide (Thermo Scientific), dihydrotestosterone (Sigma), PGE2 (Bioscience), Noggin (Thermo Scientific), and R-spondin (R & D Systems) [32]. Tumor cells were seeded in a 96-well plate with Matrigel diluted in a 1:3 ratio of ADMEM complete medium and incubated at 37 °C for 15 min to solidify the matrigel complex. Next, ADMEM complete medium mixed with PTUPB, with or without enzalutamide, was added to each well. The viability of the organoids was analyzed using the CellTiter-Glo Luminescent assay (Promega) and visualized by immunofluorescence using the LIVE/DEAD^®^ Viability/Cytotoxicity Assay Kit (Thermo Scientific) according to the manufacturer’s protocol.

### 2.13. Animal studies and treatment regimens

All animals used in this study received humane care in compliance with applicable regulations, policies, and guidelines relating to animals. All experimental procedures using animals were approved by the Institutional Animal Care and Use Committee of UC Davis. CWR22Rv1 cells (4 million) were mixed with matrigel (1:1) and injected subcutaneously into the flanks of 4–5-week-old male C.B17/lcrHsd-Prkdc-SCID mice (ENVIGO). Tumor-bearing mice (tumor volume around 50–100 mm3) were randomized into four groups (8 tumors per group) and treated as follows: (1) vehicle control (15% Cremophor EL, 82.5% PBS and 2.5% dimethyl sulfoxide (DMSO), intraperitoneal (i.p.)), (2) enzalutamide (25 mg per kg, Per os (p.o.) daily), (3) JG231 (4 mg per kg, i.p. every other day), (4) enzalutamide (25 mg per kg, p.o. daily) plus JG231 (4 mg per kg, i.p. every other day), Tumors were measured using calipers twice a week and tumor volumes were calculated using length × width × width × 0.52. Tumor tissues were harvested and weighed after 18 days of treatment. Tumor tissues were paraffin embedded and H/E stained.

### 2.14. Immunohistochemistry

Tumors were fixed by formalin and paraffin embedded tissue blocks were dewaxed, rehydrated, and blocked for endogenous peroxidase activity. Antigen retrieving was performed in sodium citrate buffer (0.01 mol per Litter, pH 6.0) in a microwave oven at 1000 W for 3 min and then at 100 W for 20 min. Nonspecific antibody binding was blocked by incubating with 10% fetal bovine serum in PBS for 30 min at room temperature. Slides were then incubated with anti-Ki67 (at 1:500; Neomarker), anti-AR (at 1:200; Santa Cruz Biotechnology) or anti-AR-V7 (at 1:200; Precision) at 4 °C overnight. Slides were then washed and incubated with biotin-conjugated secondary antibodies for 30 min, followed by incubation with avidin DH-biotinylated horseradish peroxidase complex for 30 min (Vectastain ABC Elite Kit, Vector Laboratories). The sections were developed with the diaminobenzidine substrate kit (Vector Laboratories) and counterstained with hematoxylin. Nuclear staining of cells was scored and counted in 5 different vision fields. Images were taken with an Olympus BX51 microscope equipped with DP72 camera.

### 2.15. Statistical analysis

Statistical analyses were performed using SPSS software (RRID: SCR_002865). Raw data were summarized by means, standard deviations (SD), and graphical summaries, and then transformed, if necessary, to achieve normality. Sample size was determined based on the power to detect significant differences *(p < 0.05)*. No sample or data point from the analysis was excluded. The experiments and data process were not blinded. Data are presented as mean ± SD from three independent experiments. Differences between individual groups were analyzed using a two-tailed Student’s t-test for single comparisons or one-way analysis of variance (ANOVA), followed by the Scheffé procedure for multiple group comparisons. In the tumor growth experiments, size of the tumor at sacrifice serves as the primary response measure. The tumor growth across groups was analyzed by ANOVA. A *p*-value less significance was set at *p < 0.05*.

## 3. Data availability statement

The data obtained in this study are available upon reasonable request from the corresponding author.

## 4. Results

### 4.1. JG98 suppresses prostate cancer cell growth and re-sensitizes enzalutamide treatment

**Fig. 1.**
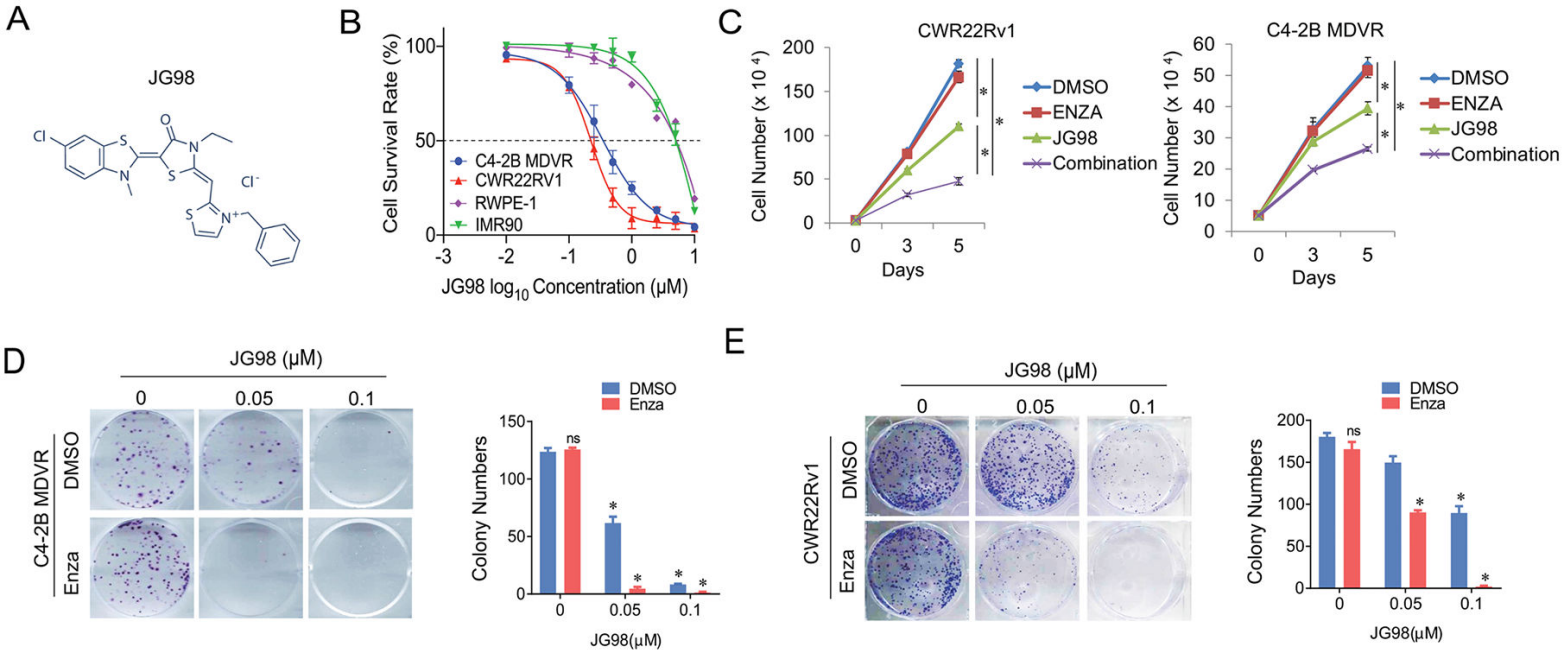
JG98 suppresses prostate cancer cell growth and re-sensitizes enzalutamide treatment A. Chemical structure of JG98. B. C4–2B MDVR, CWR22Rv1, IMR90, and RWPE-1 cells were treated with increasing doses (0.01, 0.1, 0.25, 0.5, 1, 2.5, 5, and 10 μM) of JG98 for 5 days and the viable cells were counted. The results were compared to the control to generate the cell survival rate. C. CWR22Rv1 and C4–2B MDVR cells were treated with control, 20 μM enzalutamide, 0.25 μM JG98 or the combination for 3 and 5 days, and the cell proliferation curves were plotted. D-E. 1000 C4–2B MDVR or CWR22Rv1 cells were treated with control, 0.05, 0.1 μM of JG98 in the absence or presence of enzalutamide (20 μM) and allowed to grow for 2 weeks for clonogenic assays. The colony numbers were counted for comparison. *p < 0.05. Results are the mean of three independent experiments (± S.D.). ns: not significant.

To determine whether JG98 (Fig. 1A) suppressed enzalutamide resistant prostate cancer cell growth, two resistant cell lines, C4–2B MDVR and CWR22Rv1, were treated with different doses of JG98 in FBS condition. The normal fibroblast cell line IMR90 and immortalized prostate epithelial cell line RWPE-1 were used as controls. As shown in Fig. 1B, JG98 suppressed the proliferation of both C4–2B MDVR and CWR22Rv1 cells in a dose-dependent manner. However, IMR90 and RWPE-1 cells were less sensitive to JG98 treatment. We also determined the JG98 effects in charcoal stripped FBS condition. As shown in Supplemental Fig. 1A-B, JG98 effectively suppressed both CWR22Rv1 and C4–2B MDVR cells in a dose dependent manner. Furthermore, we determined the combined effects of JG98 and enzalutamide on drug-resistant cells at different time points. As shown in Fig. 1C, both CWR22Rv1 and C4–2B MDVR cells were resistant to enzalutamide. Treatment with 0.25 μM JG98 suppressed cell growth. However, the combination of JG98 and enzalutamide further reduced cell numbers. The results were confirmed using a colony formation assay. As shown in Fig. 1D, enzalutamide treatment did not affect colony number or size in CWR22Rv1 or C4–2B MDVR cells. JG98 suppresses colony formation in a dose-dependent manner. The combination of JG98 and enzalutamide synergistically suppressed colony formation in the resistant cells. Collectively, these data suggested that the HSP70 inhibitor, JG98, suppresses the growth of enzalutamide resistant prostate cancer cells and re-sensitizes them to enzalutamide treatment.

### 4.2. JG98 degrades AR-V7 and suppresses HSP70 induced AR-V7 transcriptional activity

**Fig. 2.**
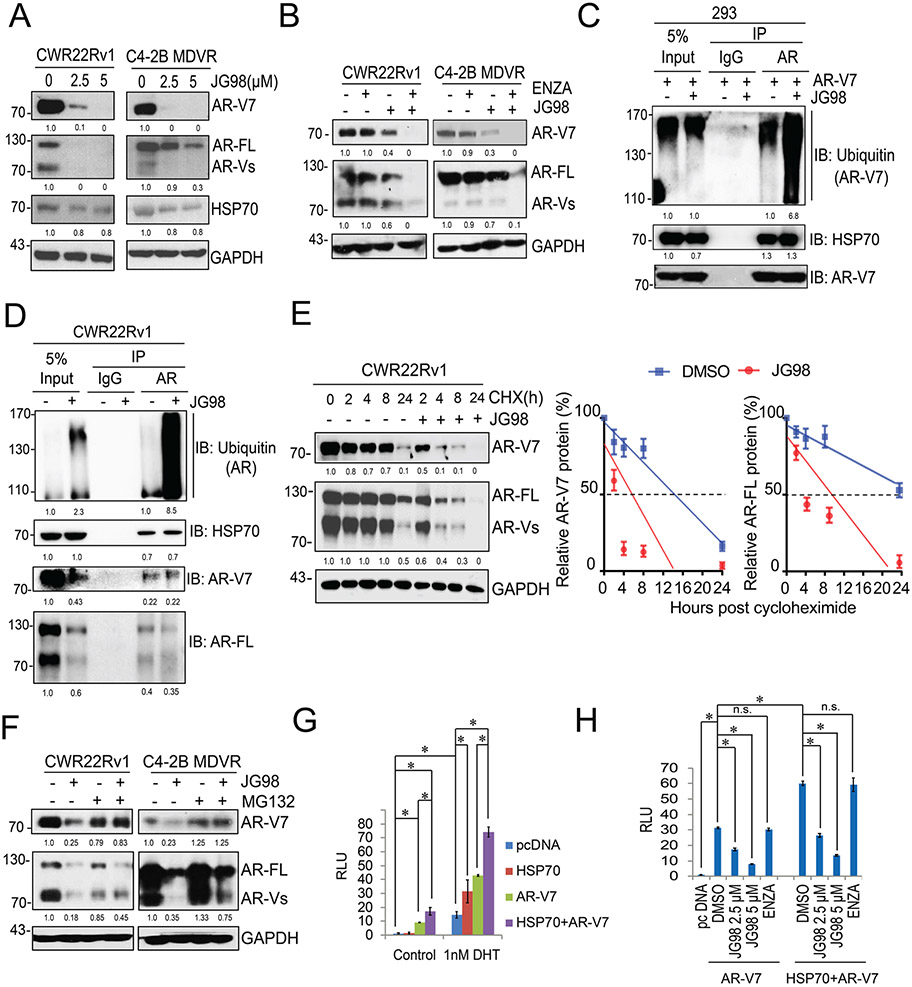
JG98 degrades AR-V7 and suppresses HSP70 induced AR-V7 transcriptional activity A. Whole cell lysates of CWR22Rv1 and C4–2B MDVR cells after 24 h treatment with JG98 (0, 2.5 or 5 μM) were separated by electrophoresis and probed for AR-V7, AR-FL, and HSP70 with their respective antibodies. GAPDH was used as the internal control. B. CWR22Rv1 and C4–2B MDVR cells were treated with DMSO, JG98 (0.25 μM), enzalutamide (20 μM), or the combination for 5 days and the levels of AR-V7 and AR-FL were examined by western blotting. GAPDH was used as the internal control. C. HEK293 cells transfected with the AR-V7 expression construct were treated with or without JG98 (2.5 μM) and immunoprecipitated with anti-AR antibodies and probed for ubiquitin, AR-V7, and HSP70, respectively. IgG antibodies were used as the negative control and whole lysate input were loaded alongside. D. CWR22Rv1 cells with the endogenous AR/AR-V7 were treated with or without JG98 (2.5 μM) for 24 h. Cell lysates were immunoprecipitated with anti-AR antibodies and probed for ubiquitin, AR-V7, AR-FL, and HSP70, respectively. E. CWR22Rv1 cells were treated with 50 μg/ml cycloheximide in the absence or presence of 5 μM of JG98. Total cell lysates were collected 0, 2, 4, 8, and 24 h after treatment. AR-V7 and AR-FL were analyzed by western blotting to calculate the half-life of AR-V7 and AR-FL. F. CWR22Rv1 and C4–2B MDVR cells were treated with or without JG98 (2.5 μM) in the absence or presence of the proteosome inhibitor, MG132 (5 μM), for additional 8 h, and the protein expression of AR-V7 and AR-FL were analyzed by western blotting. G. HEK293 cells were transiently transfected with pcDNA, HSP70, AR-V7, or the combination constructs with PSA-Luc, and treated with control or 1 nM DHT. Cell lysates were harvested 24 h after treatments, and the PSA luciferase activity was assessed. H. HEK293 cells were transiently transfected with vector only, AR-V7, or AR-V7+HSP70 expressing plasmids with PSA-Luc, and subsequently treated with DMSO, JG98 (2.5, 5 μM) or enzalutamide (20 μM). PSA luciferase activity was measured. *p < 0.05. Results are the mean of three independent experiments (± S.D.). ns: not significant.

To determine whether JG98 affected AR and AR-V7 expression, CWR22Rv1 and C4–2B MDVR cells were treated overnight with different doses of JG98. As shown in Fig. 2A, JG98 significantly suppressed AR-V7 protein expression. Notably, the full-length AR was also suppressed in these resistant cells. However, JG98 only slightly decreased HSP70 expression. Importantly, the combination of the JG98 and enzalutamide significantly inhibited the protein expression of AR and AR-V7 compared to single treatment alone (Fig. 2B). To further determine whether JG98 decreased AR-V7 expression through enhanced ubiquitination, a co-immunoprecipitation assay was performed in HEK293 and CWR22Rv1 cells. As shown in Fig. 2C and Supplemental Fig. 2A, JG98 significantly enhanced AR-V7 ubiquitination compared to DMSO-treated control cells. Notably, JG98 also promoted AR and AR variant ubiquitination in CWR22Rv1 cells in both FBS and CS-FBS condition (Fig. 2D and Supplemental Fig. 2B). We then investigated whether JG98 affected AR/AR-V7 protein stability in enzalutamide resistant CWR22Rv1 cells. The results showed that JG98 treatment significantly shortened the half-life of AR-V7 (approximately 6 h) in CWR22Rv1 cells compared with that in DMSO-treated controls (approximately 16 h). JG98 also shortened the half-life of AR-FL (Fig. 2E). To determine whether the decreased AR-V7 protein expression by JG98 treatment was mediated through the ubiquitin-proteasome pathway, the proteasome inhibitor MG132 was added to C4–2B MDVR and CWR22Rv1 cells. While JG98 decreased AR-V7 expression, addition of MG132 blunted JG98’s effects (Fig. 2F). These results indicated that JG98 regulates AR-V7 and AR-FL protein expression through the ubiquitin-proteasome pathway. Since HSP70 binds to AR-V7, we further determined the AR-V7 transcriptional activity affected by HSP70. We found that AR-V7 was constitutively active and that HSP70 significantly increased AR-V7 transcriptional activity. DHT treatment further increased HSP70-induced AR-V7 transcriptional activity (Fig. 2G). We then determined whether JG98 could block the HSP70-induced AR-V7 transcriptional activity. As shown in Fig. 2H, JG98 not only blocked AR-V7 transcriptional activity but also suppressed HSP70-induced AR-V7 transcriptional activity in a dose-dependent manner. However, enzalutamide treatment had no effect. Overall, JG98 promotes AR-V7 protein degradation and suppresses HSP70-induced AR-V7 transcriptional activity in enzalutamide resistant prostate cancer cells.

### 4.3. JG98 promotes AR-V7 degradation through STUB1

**Fig. 3.**
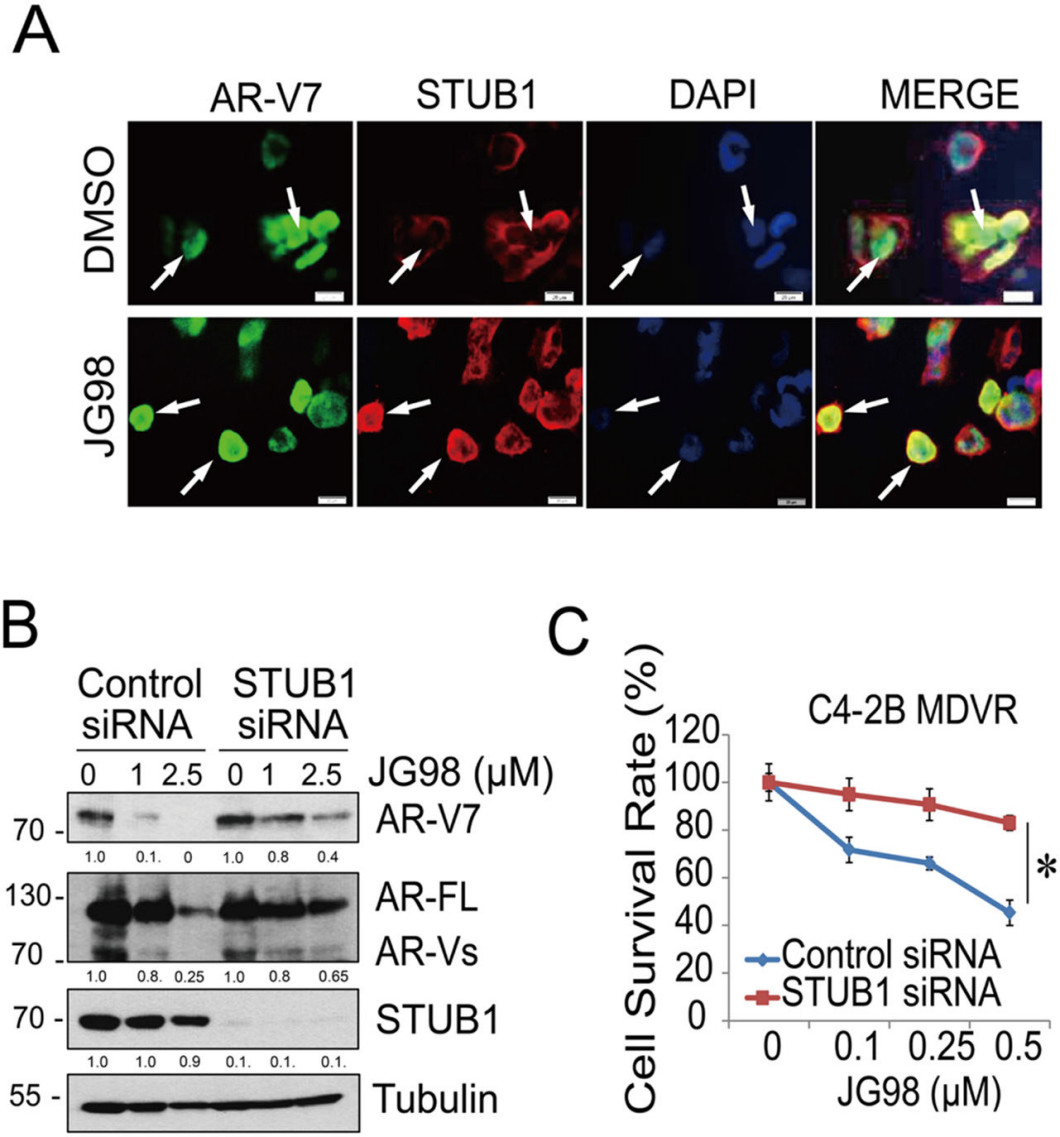
JG98 promotes AR-V7 degradation through STUB1 A. HEK293 cells were co-transfected with AR-V7, HSP70, and Flag-STUB1 for 3 days and then treated with 2.5 μM JG98 for 24 h. The cells were then permeabilized with paraformaldehyde and probed with anti-AR-V7 and anti-Flag antibodies, respectively. Location of AR-V7 was visualized with FITC-conjugated and Flag-STUB1 with Alexa467-conjugated secondary antibodies. Nuclei were stained with DAPI. Merged images displayed interaction between AR-V7 and Flag-STUB1 under JG98 treatment. White arrows indicate the typical staining of cells in each group. Scale bar 20 μm. B. C4–2B MDVR cells treated with STUB1 siRNA or control and treated with various doses of JG98 (0, 1, 2.5 μM) for 24 h. Whole cell lysates were separated by electrophoresis and blotted with AR-V7, AR-FL and STUB1 antibodies. Levels of tubulin were assessed for loading equity. C. C4–2B MDVR cells were transfected with siRNA against STUB1 or control for 5 days. Cell viability was determined by cell counting and represented as cell survival rates. *p < 0.05. Results are the mean of three independent experiments (± S.D.).

To determine whether JG98-induced AR-V7 protein degradation occurred through STUB1, we performed dual immunofluorescence staining experiments in HEK293 cells. As shown in Fig. 3A, STUB1 and AR-V7 did not colocalize in cells when HSP70 was present; AR-V7 was predominantly expressed in the nucleus, whereas STUB1 was mostly localized in the cytoplasm. The “donut shaped” staining pattern indicates that HSP70 may prevent STUB1 from translocating into the nucleus and binding to AR-V7. JG98 treatment significantly promoted STUB1 nuclear translocation and colocalization with AR-V7. Notably, we found that STUB1 knockdown in C4–2B MDVR cells partially rescued AR and AR-V7 suppression by JG98 treatment. As shown in Fig. 3B, JG98 suppressed AR-V7 expression in a dose-dependent manner. However, STUB1 significantly diminished JG98 effects on AR and AR-V7 protein expression levels. It is known that other E3 ligases mediate AR degradation [33], which is likely why some degradation still occurred after STUB1 knockdown. The importance of STUB1 in the process was also tested by measuring effects on cell proliferation. As shown in Fig. 3C, JG98 suppressed the growth of C4–2B MDVR cells in a dose-dependent manner. However, STUB1 knockdown significantly reduced JG98 growth inhibition effects. Collectively, these data suggested that AR/AR-V7 degradation induced by JG98 is mediated primarily by STUB1.

### 4.4. JG98 regulates gene programs in enzalutamide resistant prostate cancer cells

**Fig. 4.**
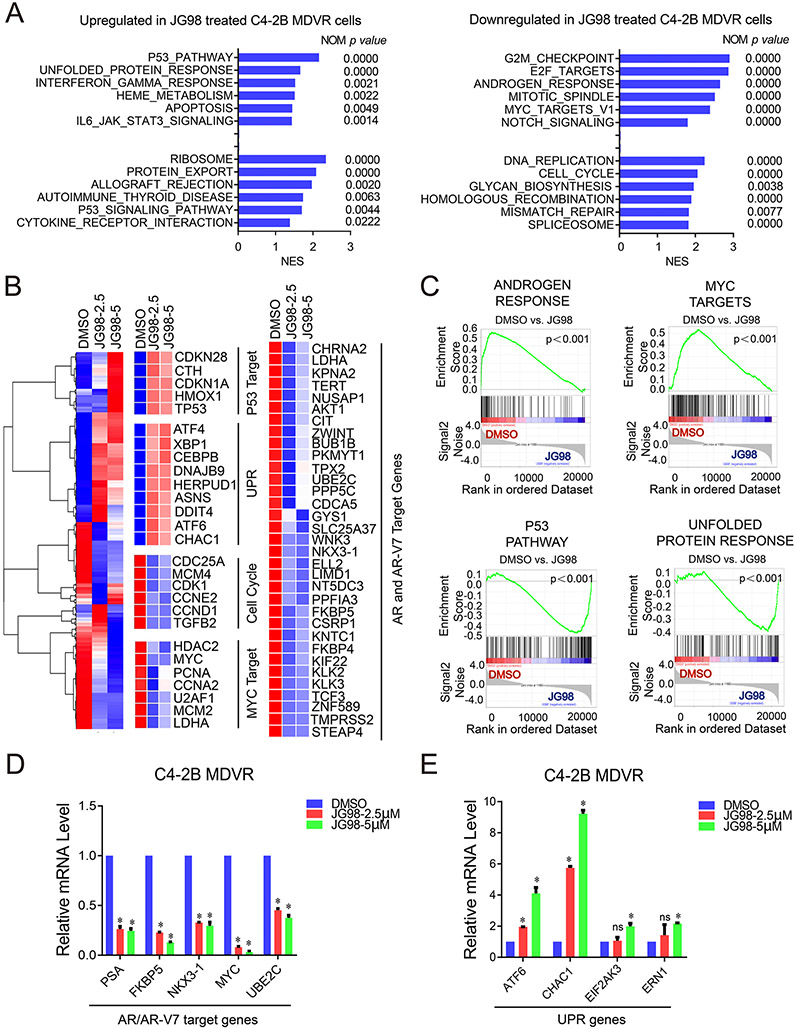
JG98 regulates gene programs in enzalutamide resistant prostate cancer A. GSEA of top enriched gene sets in C4–2B MDVR cells treated by JG98. The upregulated and downregulated gene sets from the Hallmark and KEGG platforms were outputted by GSEA. B. Heatmap and hierarchical clustering of the differentially expressed genes (DEGs) between treatments (JG98 2.5 μM and JG98 5 μM) in C4–2B MDVR cells with FPKM> 1 and log2 fold change > 0.25, as compared to vehicle (DMSO). The genes were displayed in rows and the normalized counts per sample were displayed in columns. Red indicates upregulated, and blue designates downregulated expression levels. Right: P53 target, UPR, Cell Cycle, Myc target, and AR/AR-V7 target genes that were altered in expression are displayed. C. GSEA of the gene signatures up or down regulated in C4–2B MDVR cells treated with JG98, as compared to DMSO. The signature was defined by genes that are preferentially downregulated in the androgen response and Myc target pathways. GSEA of the P53 pathway and unfolded protein response signatures were upregulated by JG98. D. qRT-PCR analysis of the indicated genes from the AR and AR-V7 target and UPR pathways in C4–2B MDVR cells treated with DMSO or JG98 (2.5 or 5 μM) for 24 h. *p < 0.05, Results are the mean of three independent experiments (± S.D.). ns: not significant.

To further explore the gene regulatory mechanisms underlying the downregulation of HSP70 in drug-resistant prostate cancer cells, we performed RNA sequencing using C4–2B MDVR cells treated with JG98 to identify the gene programs affected by the treatment. In total, 12,663 genes were differentially expressed in JG98-treated C4–2B MDVR cells. The top pathways upregulated by JG98 treatment included the p53 pathway, unfolded protein response (UPR), and the apoptosis pathway. The downregulated pathways included androgen response, Myc targets, cell cycle, and E2F targets as analyzed by GSEA (Fig. 4A). Heatmaps drawn using hierarchical clustering showed that, compared with the DMSO group, the JG98-treated groups had significant differences in gene expression, and the expression changes of these genes induced by different concentrations of JG98 treatment was consistent (Fig. 4B left). At the individual gene level, we observed upregulation of P53 target genes (CDKN1A, CDKN2B, CTH, and TP53) and UPR genes (CHAC1, ATF4, and ATF6) in JG98-treated cells. In contrast, JG98 treatment significantly inhibited AR and AR-V7 target genes (AKT1, UBE2C, and NKX3–1), cell cycle genes (CDC25A, MCM4, and CDK1), and Myc target genes (MYC, PCNA, and MCM2). We also found a significant enrichment of androgen response and Myc target signaling in the DMSO-treated group by GSEA. However, the genes from the P53 and UPR pathways were significantly enriched in the JG98-treated groups (Fig. 4C). qRT-PCR determined if JG98 affect mRNA expression of AR and AR variants in C4–2B MDVR cells (Supplementary Fig. 3). Intriguingly, JG98 treatment did not affect the levels of AR-V1 and AR-V7 mRNA, but significantly decreased the levels of AR-FL, AR-V3, AR-V4, and AR-V9 mRNA. These results suggested that JG98 may alter AR-FL and some AR variant expression at both mRNA and protein levels. We also verified that AR and AR-V7 target genes, such as PSA, NKX3–1, FKBP5, UBE2C, and Myc, were suppressed by JG98 treatment (Fig. 4D left). Importantly, genes such as ATF6, CHAC1, EIF2AK3, and ERN1, which belong to the UPR signaling pathway, were significantly up regulated by JG98 treatment (Fig. 4D right). Taken together, these results suggested that JG98 treatment regulates gene programs in enzalutamide resistant prostate cancer cells.

### 4.5. JG98 improves enzalutamide treatment in clinically relevant CRC and PDX organoid models

**Fig. 5.**
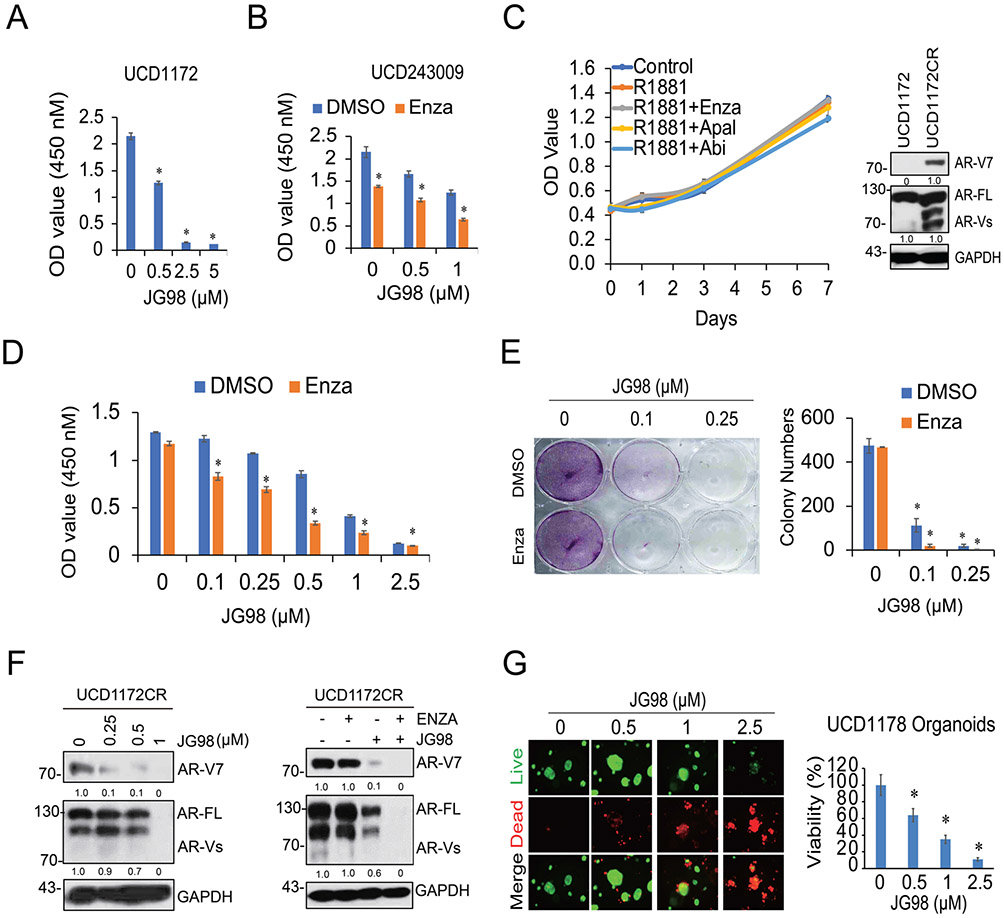
JG98 improves enzalutamide treatment in CRC and PDX organoid models A. CRCs derived from UCD1172 PDX tumors were treated with various doses of JG98 for 5 days, and the cell growth was determined by the CCK-8 assay. B. CRCs from UCD243009 PDX tumors were treated with JG98 alone or in combination with enzalutamide (20 μM) for 5 days, and the cell proliferation was assayed by CCK-8. C. UCD1172CR cells were seeded in charcoal stripped FBS medium and treated with 10 nM R1881 alone or in combination with antiandrogens (enzalutamide, abiraterone or apalutamide). Cell growth was monitored over 7 days by the CCK-8 assay. Whole cell lysates from UCD1172 and UCD1172CR were separated by electrophoresis. The status of AR-V7 and AR-FL was shown by western blotting. D. UCD1172CR cells were treated with increasing concentrations of JG98 (0, 0.1, 0.25, 0.5, 1, 2.5 μM) in the absence or presence of enzalutamide (20 μM) for 3 days. Cell viability was determined by the CCK-8 assay. E. UCD1172CR cells were treated with JG98 alone or with enzalutamide (20 μM) in the clonogenic assay. The number of colonies in each condition was counted and plotted. F. UCD1172CR cells were treated with DMSO or JG98 (0.25, 0.5, 1 μM) for 5 days and the cell lysates were analyzed for the expression of AR-V7 and AR-FL by western blotting (left). For the combination treatment, these cells were treated with DMSO, JG98 (0.25 μM), enzalutamide (20 μM), and JG98 +enzalutamide for 5 days. Protein expression was examined by western blotting (right). G. Organoids from UCD1178 PDX were treated with JG98 alone or together with enzalutamide (20 μM) for 7 days. Cell viability was assayed by CellTiter-Glo Luminescent assay and the live-and-dead cells were visualized by immunofluorescence. *p < 0.05. Results are the mean of three independent experiments (± S.D.).

We successfully generated multiple CRCs, PDX, and organoid models from patients with advanced prostate cancer. In this study, we determined the effects of JG98 in these models. We first tested two CRCs generated from advanced prostate tumors, UCD1173 and UCD1172, from an enzalutamide-failed patient and a Gleason score 10 patient, respectively. As shown in Fig. 5A and Supplementary Fig. 4A, JG98 suppressed both the CRCs in a dose-dependent manner. We further tested the combined effects of JG98 and enzalutamide in additional CRCs. As shown in Fig. 5B and Supplementary Fig. 4B, enzalutamide and JG98 treatment slightly suppressed cell growth, whereas JG98 combined with enzalutamide further reduced cell growth in UCD243009 and UCD1177. We also developed a novel castration-resistant CRC cell line from UCD1172 by performing multiple tumor implantations in castrated mice, and subsequently regrowing the cells *in vitro*. As shown in Fig. 5C left, UCD1172CR cells were not responsive to androgen treatment and were resistant to ARSI treatment. Western blotting confirmed that AR-V7 was highly overexpressed in the UCD1172CR cells (Fig. 5C right). JG98 synergistically enhanced enzalutamide treatment of UCD1172CR cells in a dose-dependent manner (Fig. 5D). The results were confirmed by a colony formation assay (Fig. 5E). Similar to the enzalutamide resistant cell line data, JG98 treatment significantly decreased AR-V7 and AR-FL expression in UCD1172CR cells (Fig. 5F). We also determined the effects of JG98 in two organoid models, UCD1173 and UCD1178, which were generated from the tumors of patients with enzalutamide failure. As shown in Fig. 5G and Supplementary Fig. 4C, JG98 induced the death of organoids in both models in a dose-dependent manner. Collectively, these data further determined the effects of JG98 in clinically relevant CRC and organoid models.

### 4.6. JG231, the JG98 analog, inhibits AR-V7 and improves enzalutamide treatment in vitro and in vivo

**Fig. 6.**
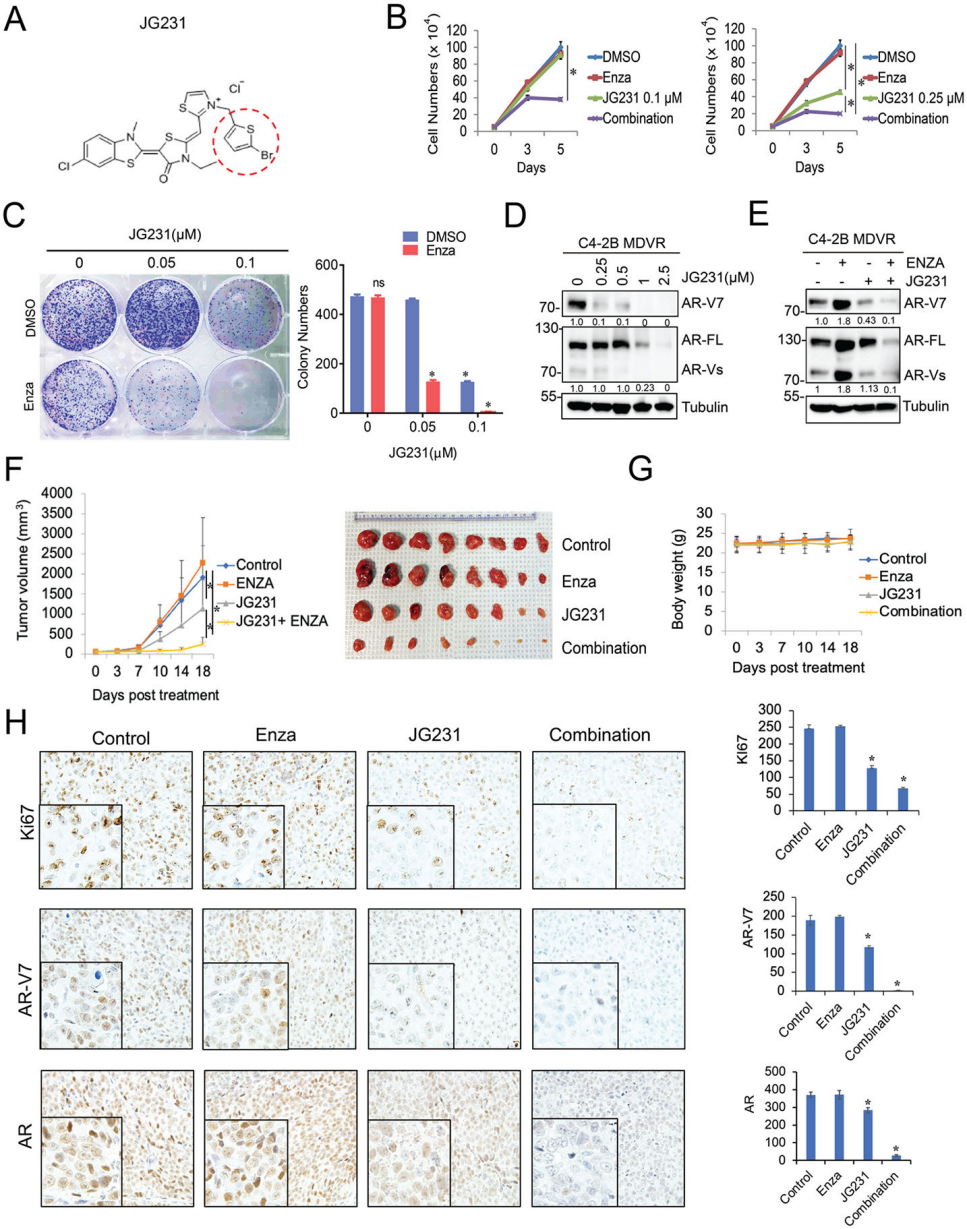
JG231, the JG98 analog, inhibits AR-V7 and improves ARSI treatment *in vitro* and *in vivo*. A. The chemical structure of JG231 B. C4–2B MDVR cells were treated with JG231 (0.1 or 0.25 μM) alone or in combination with enzalutamide (20 μM), and cell viability was measured by cell counting at different time points. C. Clonogenic assay was performed with C4–2B MDVR cells treated with JG231, with or without enzalutamide (20 μM). Colonies were stained by crystal violet and the number was counted. D. C4–2B MDVR cells were treated with increasing concentrations of JG231 (0, 0.25, 0.5, 1, 2.5 μM) for 3 days and the cell lysates were evaluated for AR-V7 and AR-FL expression by western blotting. E. C4–2B MDVR cells were treated with DMSO, enzalutamide (20 μM), JG231 (0.25 μM), or the combination for 5 days, and the expression of AR-V7 and AR were determined by western blotting. F-G. Mice bearing CWR22Rv1 xenografts were treated with vehicle control, enzalutamide (25 mg/Kg p.o), JG231 (4 mg/Kg i.p), or JG231 plus enzalutamide for 18 days (n = 8). Tumor volumes were measured twice weekly. Tumors were photographed and weighed. Data represent means ± S.D. from 8 tumors per group. H. IHC staining of Ki67, AR-V7, and AR in each group was performed. *p < 0.05, Results are the mean of three independent experiments (± S.D.).

We further investigated the newly synthesized HSP70 inhibitor, JG231, which was modified from JG98. Instead of the benzyl group found in JG98, JG231 contains 2-Bromothiophene, making it more potent when binding with HSP70 (Fig. 6A). This replacement dramatically improves the solubility of the drug and reduces its toxicity. Moreover, JG231 has superior pharmacokinetic characteristics, with a two-fold increase in C_max_ and T_max_ compared to those of JG98 [23]. Therefore, JG231 worth to be further investigated in drug-resistant models. Here, we showed that JG231 effectively synergized with enzalutamide treatment in C4–2B MDVR cells (Fig. 6B). 0.1 μM JG231 and 20 μM enzalutamide had minimal effects on the proliferation of C4–2B MDVR cells. However, combination treatment significantly suppressed cell growth at different time points. Single treatment with 0.25 μM JG231 suppressed cell proliferation, while adding enzalutamide completely inhibited cell growth. These results were confirmed by colony formation assay. As shown in Fig. 6C, JG231 suppressed C4–2B MDVR colony formation in a dose-dependent manner. Combination with enzalutamide further reduced colony size and numbers. Notably, JG231 synergistically enhanced all ARSI treatments in C4–2B MDVR cells (Supplementary Fig. 5A). Similar to JG98, JG231 suppressed AR-V7 expression in a dose-dependent manner at different time points (Fig. 6D and Supplementary Fig. 5B). Importantly, JG231 blocked enzalutamide-induced AR-V7 expression in C4–2B MDVR cells (Fig. 6E). To examine whether JG231 enhanced enzalutamide treatment *in vivo*, we generated enzalutamide resistant xenografts derived from CWR22Rv1 cells. CWR22Rv1 tumors were resistant to enzalutamide treatment and JG231 significantly inhibited tumor growth. The combination of JG231 and enzalutamide further inhibited tumor growth in CWR22Rv1 xenografts (Fig. 6F). The treatments did not affect mouse body weights (Fig. 6G). Immunohistochemical staining of AR-FL, AR-V7, and Ki67 showed that AR-V7 expression and cell proliferation were significantly inhibited by JG231 treatment alone and further inhibited by combination treatment (Fig. 6H). Moreover, we tested JG231 in clinically relevant CRCs and organoid models. JG231 improved enzalutamide treatment in UCD1172CR cells (Supplementary Fig. 5C) and suppressed UCD1178 organoid growth in a dose-dependent manner (Supplementary Fig. 5D). Collectively, JG231 suppresses the growth of enzalutamide resistant xenograft tumors, patient derived CRC cells, and organoids.

## 5. Discussion

Reactivation of AR signaling and elevated expression of AR-V7 confer resistance to ARSI therapy in CRPC patient and are associated with a poor prognosis [11]. Therefore, there is an urgent need to develop new drugs that target AR and AR-V7 to address the current treatment dilemma. The HSP70/STUB1 complex plays a key role in the degradation of AR and their variants, especially AR-V7 [15]. In addition, proteostasis can be modulated by the inhibition of HSP70 and it provides a valuable strategy to overcome ARSI resistance. In this study, we focused on the effects of two novel HSP70 inhibitors, JG98 and JG231, on cell proliferation and AR/AR-V7 expression in CRPC models. We found that both JG98 and JG231 suppressed the growth of enzalutamide resistant CRPC cells and re-sensitized these cells along with clinically relevant CRC and organoid models to enzalutamide treatment. JG98 degraded AR-V7 and suppressed HSP70-induced AR-V7 transcriptional activity in enzalutamide resistant CRPC cells. Mechanistically, JG98 inhibited AR-V7 and AR-FL protein expression *via* the ubiquitin-proteasome pathway, and the degradation of these two proteins induced by JG98 was mediated by STUB1. We also explored the gene regulatory mechanisms underlying the JG98 treatment in C4–2B MDVR cells using RNA sequencing. JG98 treatment downregulated the androgen response, cell cycle pathway, and the target genes of AR/AR-V7, Myc, and E2F. It can also upregulate the UPR, p53, and apoptosis pathways. These preclinical findings set the foundation for future development of HSP70-targeting drugs to treat CRPC.

The HSP70s family includes constitutively expressed members HSC70 (HSPA8) and stress-inducible member HSP70 (HSPA1A/HSPA1B), which have been demonstrated to control protein maturation and proper folding in cancer cells. These chaperone proteins regulate the activity and stability of many oncogenes that control cancer cell survival and progression [34]. In prostate cancer, HSP70 plays an important role in inhibiting cell apoptosis, cell cycle regulation, metastasis, and AR transcriptional activity and stability [35]. Studies have shown that serum HSP70 expression is significantly elevated in patients with prostate cancer [36]. Furthermore, CRPC is associated with an increased dependence on HSP70 [37], and GRP78 expression is significantly elevated in metastatic CRPC compared to localized prostate cancer [38]. Moreover, GRP75 expression is associated with an increased risk of high-grade prostate cancer [39]. These findings suggest that HSP70 may serve as a diagnostic and prognostic marker for prostate cancer. Mechanistically, accessory chaperone STUB1, a functional E3 ubiquitin ligase, links the polypeptide-binding activity of HSP70 to the ubiquitin-proteasome system. Specifically, while HSP70 prevents the correct folding of substrate proteins, the recruitment of STUB1 simultaneously promotes the U-box-dependent ubiquitination of these HSP70-bound substrates [40]. Previous studies have shown that AR and its variant, AR-V7, are targets of the HSP70/STUB1 complex. STUB1 blocks the formation of the HSP70 and AR/AR-V7 complexes, resulting in AR/AR-V7 protein degradation [15]. In the present study, this conclusion was further strengthened, as we performed studies with two HSP70 allosteric inhibitors and found that they promoted STUB 1/AR-V7 co-localization and enhanced AR-V7 ubiquitination. Importantly, knockdown of STUB1 significantly diminished JG98 effects on AR-V7 protein expression and cell proliferation. These results suggested that AR/AR-V7 degradation, induced by JG98 and JG231, is mediated by STUB1. Modulation of the HSP70/STUB1 machinery overcomes antiandrogen resistance *via* the regulation of AR-V7.

Proteostasis represents an equilibrium in the overall rates of protein folding, transport, and degradation, and is maintained by a network of factors, including molecular chaperones, ubiquitin-proteasome components, and autophagy systems (40). Molecular chaperones, including heat shock proteins, are involved in controlling the stability and function of client proteins, preventing the aggregation of misfolded proteins, and degrading severely damaged proteins (41). The conversion from androgen sensitive prostate cancer to CRPC is associated with metabolic reprogramming and, therefore, these cells may require distinct proteostasis networks [41]. In addition to HSP70 and STUB1, AR is a client protein that interacts with a set of well-designed chaperones, including HSP90 and HSP40, as well as accessory chaperones, such as p23, FKBP-52, and α-SGT proteins [42]. Indeed, HSP90 inhibitors have been shown to promote AR degradation in prostate cancer cells and synergize with ADT [43], but these compounds are less effective in CRPC driven by AR-Vs signaling, such as in C4–2B MDVR and CWR22Rv1 cells [44,45]. This is important because HSP90 inhibitors demonstrated unsatisfactory results in prostate cancer clinical trials [46,47]. In contrast, HSP70 inhibitors reduce the stability of AR variants and exhibit antiproliferative activity in these CRPC cells. This phenomenon may be partly explained by differences in the molecular recognition of AR and its variants. Specifically, HSP70, but not HSP90, binds to the N-terminal motif retained in AR-Vs in CWR22Rv1 cells [48,49]. Additionally, AR variant function is independent of the HSP90 chaperone system and HSP90 inhibition induces AR variants expression [50]. Consistently, literature also showed that HSP90 inhibition may increase HSP70 expression [51]. Thus, HSP70 represents an attractive target in CRPC treatments, and HSP70 inhibitors may have the synergistic benefit of modulating signaling and transcriptional networks associated with HSP client proteins in CRPC cells [52]. Unfortunately, as the only HSP70 inhibitor for the treatment of cancer patient in clinical trials, MKT-077 is limited owing to its rapid metabolism and nephrotoxicity [19]. To address this problem, great effort has been made to develop MKT-077 analogs. JG98 was designed to improve binding affinity and metabolic stability, and this compound has been shown to induce apoptosis in breast cancer cell lines with a microsomal half-life of at least seven times higher than that of MKT-077 (51). Notably, the binding site of MKT-077 analogs, such as JG98, is highly conserved among the major human HSP70 paralogs (HSC70/HSPA8, HSP72/HSPA1, BiP/HSPA5, mtHSP70/HSPA9), suggesting that these compounds may have broad activity against various HSP70 family members [23]. However, differences in subcellular localization may dictate some selectivity for HSP70 family members [53]. In our study, we also tested JG231, an MKT-077 analog with further improved microsomal stability and better pharmacokinetic properties [54]. This compound has been shown to significantly inhibit the growth of medullary thyroid carcinoma xenografts (TT and MZ-CRC-1 cells) after intraperitoneal administration [55]. In this study, we demonstrated for the first time that JG98 and JG231 synergistically improved enzalutamide treatment in different drug resistant CRPC models. Additionally, drug-resistant CRCs and organoids were highly sensitive to JG98 and JG231 treatment. In addition to its inhibitory effect on the AR/AR-V7 signaling pathway, the treatment also activates the UPR, P53, and apoptosis pathways, but inhibits the cell cycle and Myc signaling pathways. The UPR was recently found to be upregulated after HSP70 inhibition in K562 cells [33], supporting our findings. Since HSP70 acts as a chaperone for multiple oncogenic proteins, inhibition of HSP70 may also inhibit prostate cancer cell proliferation through an AR-independent mechanism, which is a hypothesis that should be further investigated in future studies.

## 6. Conclusions

Our study demonstrates that HSP70 inhibitors and AR blockers have a synergistic inhibitory effect on drug-resistant CRPC growth, suggesting that the simultaneous inhibition of chaperone activity and AR-dependent signaling may enhance the inhibitory effect of ARSI treatment in CPRC. Modulation of HSP70/STUB1 by the novel small molecules JG98 and JG231 may be a valuable strategy for treating AR-V7-overexpressing CRPC and improving enzalutamide therapy.

## Supplementary Material

Supplemental file

## Data Availability

Data will be made available on request.
