# Supplementary material for: Allosteric inhibition of HSP70 in collaboration with STUB1 augments enzalutamide efficacy in antiandrogen resistant prostate tumor and patient-derived models
Source: Pharmacol Res. Author manuscript; Available in PMC 2023 May 5. (PMC10162009; doi:10.1016/j.phrs.2023.106692)
Supplement: Supplemental file [file NIHMS1892140-supplement-Supplemental_file.docx]

**Supplementary Information for**

**Allosteric inhibition of HSP70 in collaboration with STUB1 augments enzalutamide efficacy in antiandrogen resistant prostate tumor and patient-derived models**

Pengfei Xu, Joy C. Yang, Shu Ning, Bo Chen, Christopher Nip, Qiang Wei, Liangren Liu, Oleta T. Johnson, Allen C. Gao, Jason E. Gestwicki, Christopher P. Evans, Chengfei Liu*

*Corresponding author: Chengfei Liu

**Email:**  [cffliu@ucdavis.edu](mailto:cffliu@ucdavis.edu)

**This PDF file includes:**

Figures S1 to S5

Table S1

**Figure S1**


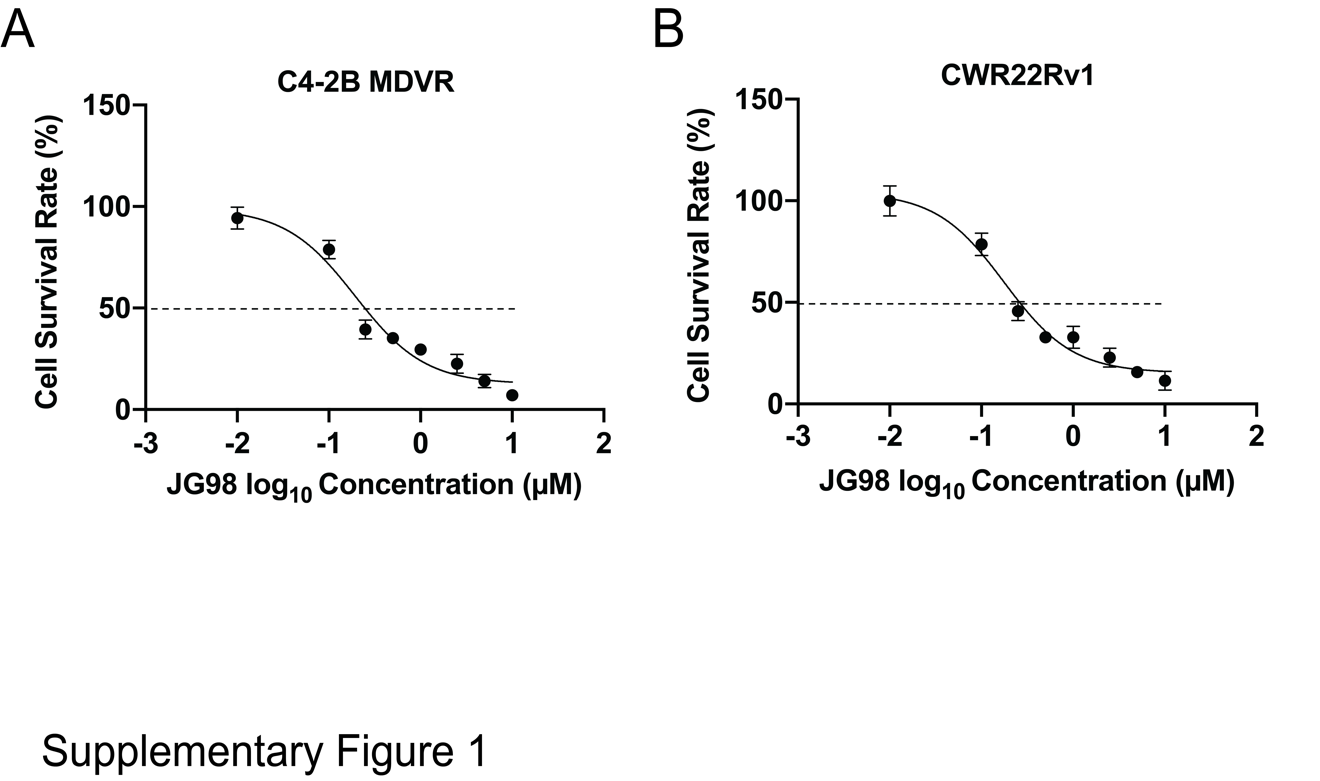


Figure S1. JG98 suppresses ARSI-resistant cells in charcoal stripped FBS condition. A-B. C4-2B MDVR and CWR22Rv1cells were cultured in charcoal stripped FBS condition and treated with increasing doses (0.01, 0.1, 0.25, 0.5, 1, 2.5, 5, and 10 µM) of JG98 for 3 days and the viable cells were counted. The results were compared to the control to generate the cell survival rate.

**Figure S2**


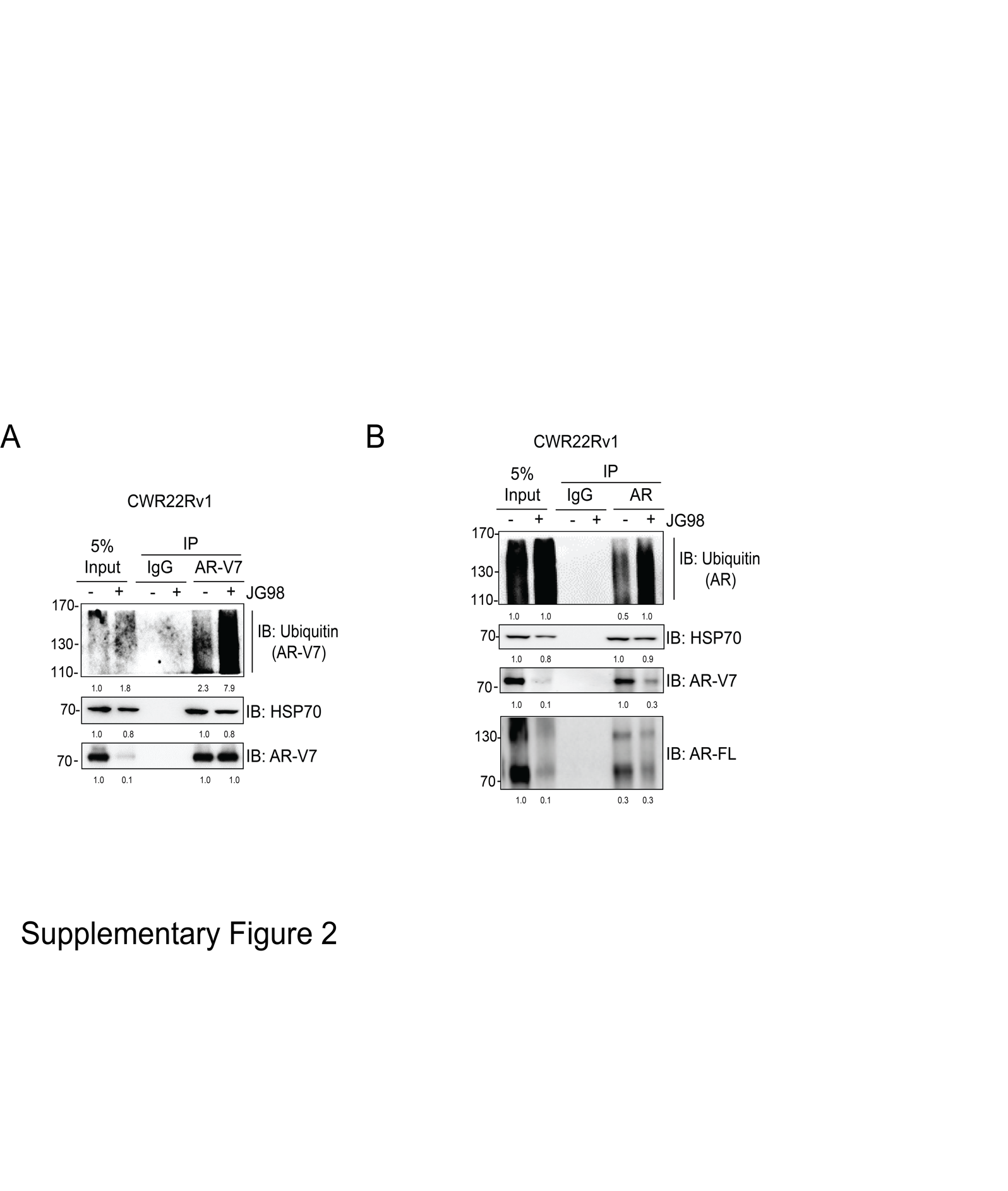


Figure S2. JG98 promotes AR/AR-V7 ubiquitination in charcoal stripped FBS condition. A. CWR22Rv1 cells were cultured in charcoal stripped FBS condition and treated with or without JG98 (2.5 µM) for 24 hours. Cell lysates were immunoprecipitated with anti-AR-V7 antibody and probed for ubiquitin, AR-V7, and HSP70, respectively. B. CWR22Rv1 cells were cultured in charcoal stripped FBS condition and treated with or without JG98 (2.5 µM) for 24 hours. Cell lysates were immunoprecipitated with anti-AR antibody and probed for ubiquitin, AR, AR-V7, and HSP70, respectively.

Figure S3


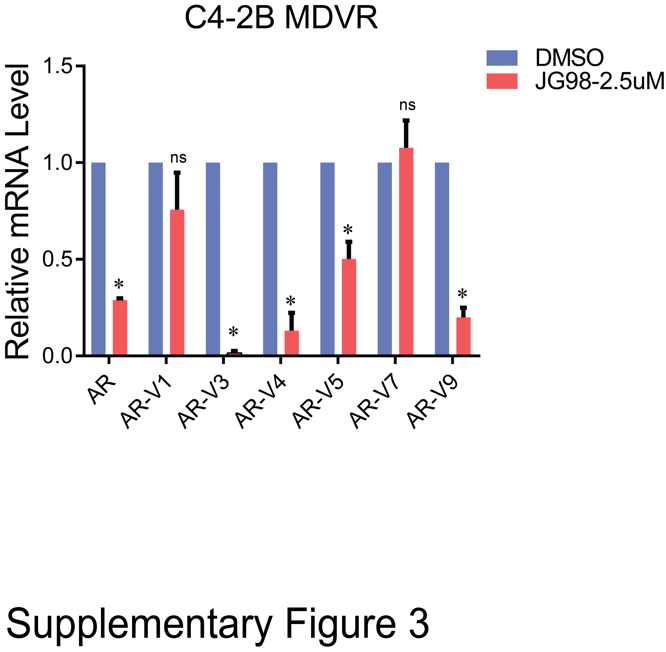


Figure S3. JG98 regulates mRNA expression of AR and AR variant. C4-2B MDVR cells were treated with 2.5 µM JG98 for 24 hours, total RNA was extracted from the cells and mRNA levels of AR-FL, AR-V1, AR-V3, AR-V4, AR-V5, AR-V7, and AR-V9 was determined by qRT-PCR. ** p<0.05.* Results are the mean of three independent experiments (±S.D.).

Figure S4


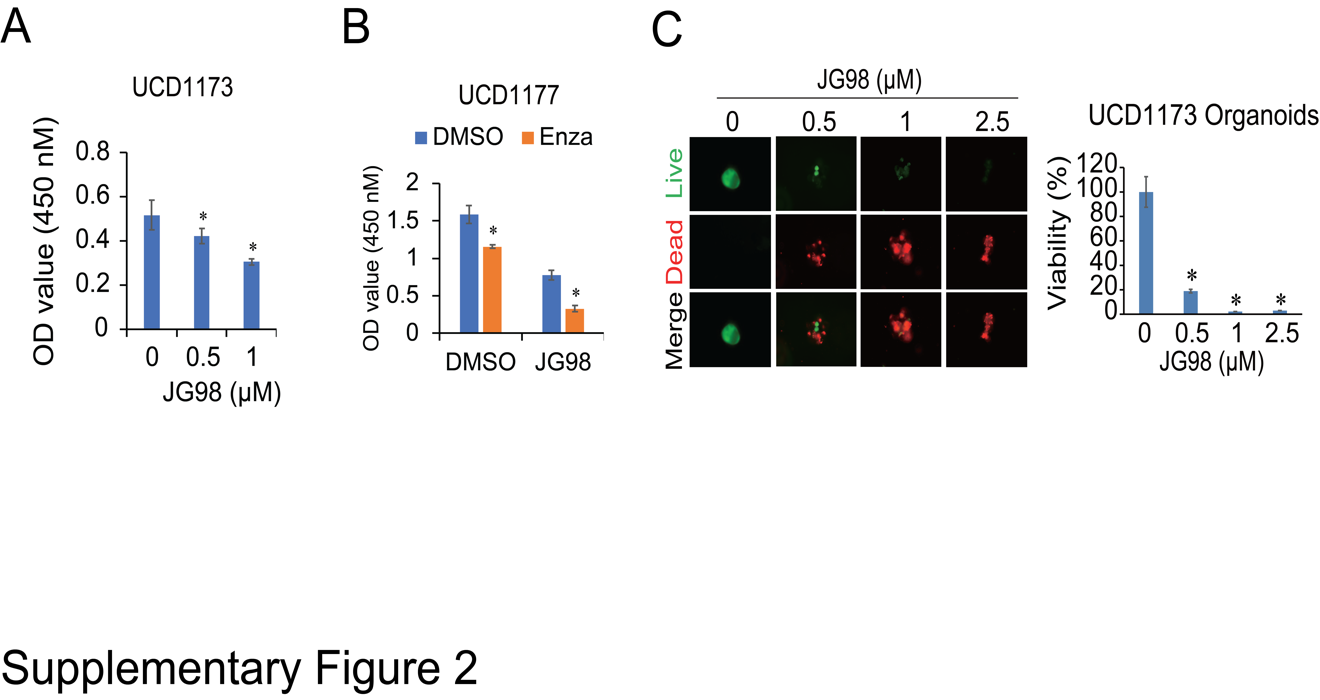


**Figure S4. JG98 improves enzalutamide treatment in CRC and PDX organoid models.** **A.** CRCs derived from UCD1173 PDX tumors were treated with various doses of JG98 for 5 days, and the cell growth was determined by the CCK-8 assay. **B.** CRCs from UCD1177 PDX tumors were treated with JG98 alone or in combination with enzalutamide (20 µM) for 5 days, and the cell proliferation was assayed by CCK-8. **C.** Organoids from UCD1173 PDX were treated with JG98 alone or together with enzalutamide (20 µM) for 7 days. Cell viability was assayed by CellTiter-Glo Luminescent assay and the live-and-dead cells were visualized by immunofluorescence. * *p<0.05.* Results are the mean of three independent experiments (±S.D.).

Figure S5


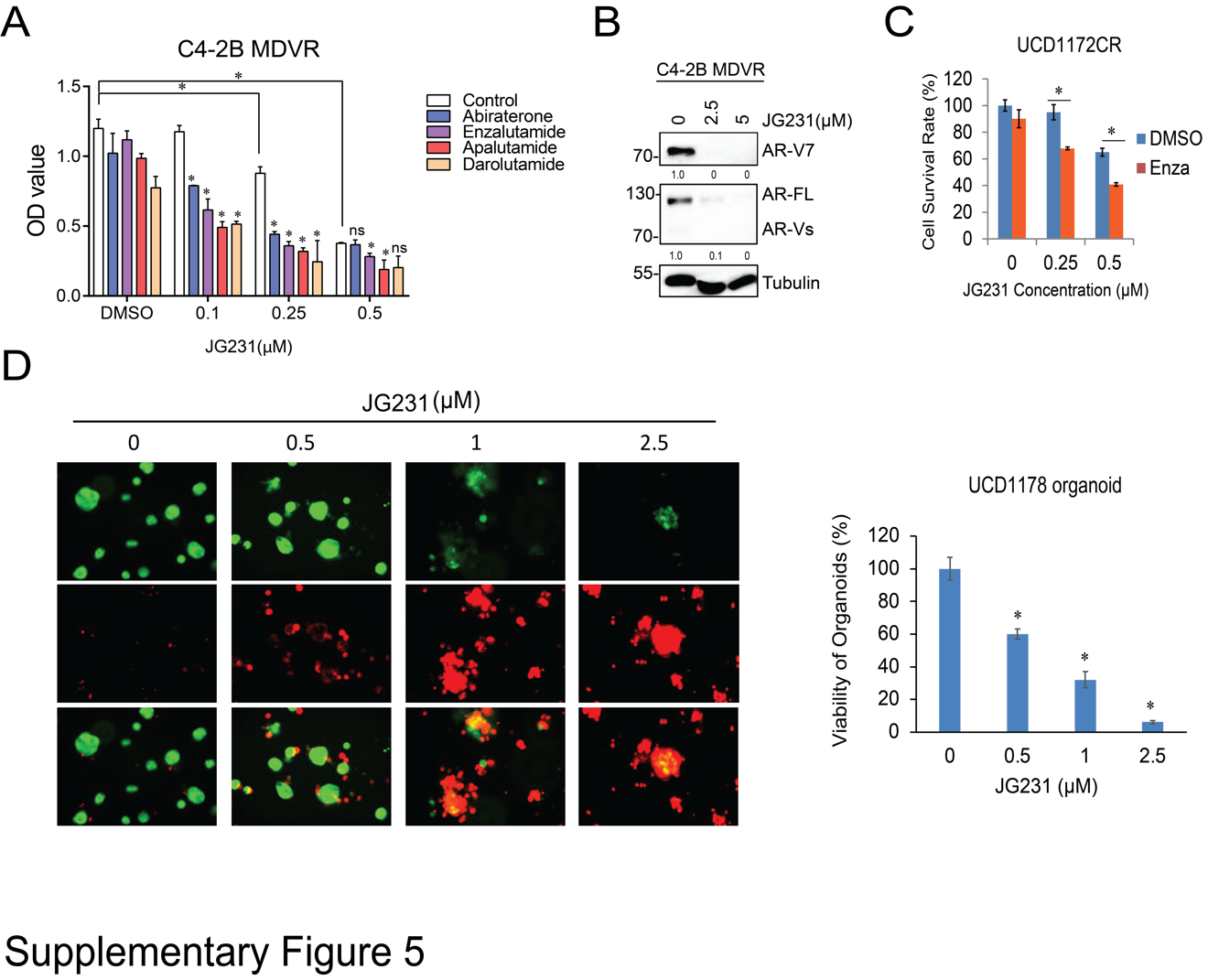


Figure S5. JG231 re-sensitizes ARSI treatment in resistant cell line, CRC, and PDX organoid models. A. C4-2B MDVR cells were treated with JG231 (0, 0.1, 0.25 µM) alone or in combination with abiraterone acetate (5 µM), enzalutamide (20 µM), apalutamide (20 µM) or darolutamide (5 µM) for 7 days, and cell viability was determined by the CCK-8 assay. B. C4-2B MDVR cells were treated with increasing concentrations of JG231 (2.5 and 5 µM) for 24 hours and the cell lysates were evaluated for AR-V7 and AR-FL expression by western blotting. C. UCD1172CR cells were treated with JG231 (0.25, 0.5 µM), enzalutamide (20 µM), or the combination for 3 days, and the effect on cell proliferation was represented by the cell survival rates. D. Organoids from UCD1178 PDX were treated with JG231 (0, 0.5, 1, 2.5 µM) for 7 days and analyzed by the CellTiter-Glo Luminescent assay, and the live-and-dead cells were visualized by immunofluorescence. * *p<0.05.* Results are the mean of three independent experiments (±S.D.).

Table S1. Primer list for q-PCR

|  | Forward | Reverse |
| --- | --- | --- |
| AR-FL | AAGCCAGAGCTGTGCAGATGA | TGTCCTGCAGCCACTGGTTC |
| AR-V1 | AACAGAAGTACCTGTGCGCC | TGAGACTCCAAACACCCTCA |
| AR-V3 | TGGATGGATAGCTACTCCGG | GTTCATTCTGAAAAATCCTTCAGC |
| AR-V4 | AACAGAAGTACCTGTGCGCC | TTCTGTCAGTCCCATTGGTG |
| AR-V5 | AACAGAAGTACCTGTGCGCC | TATGACACTCTGCTGCCTGC |
| AR-V7 | AACAGAAGTACCTGTGCGCC | TCAGGGTCTGGTCATTTTGA |
| AR-V9 | TGCGCCAGCAGAAATGATTG | GCAGCTGCTCAGGTAAGTTG |
| AR-V1 | AACAGAAGTACCTGTGCGCC | TGAGACTCCAAACACCCTCA |
| KLK3 | GCCCTGCCCGAAAGG | GATCCACTTCCGGTAATGCA |
| FKBP5 | GGGAAGATAGTGTCCTGGTTAG | GCAGTCTTGCAGCCTTATTC |
| NKX3-1 | CCGAGACGCTGGCAGAGACC | GCTTAGGGGTTTGGGGAAG |
| UBE2C | TGGTCTGCCCTGTATGATGT | AAAAGCTGTGGG GTTTTTCC |
| Myc | TGAGGAGACACCGCCCAC | CAACATCGATTTCTTCCTCATC |
| ATF6 | ACCCGTATTCTTCAGGGTGC | CACTCCCTGAGTTCCTGCTG |
| CHAC1 | CGTGGCATACCAAGTGCAAG | TGCCTTCAGTGGTTGGTCAG |
| EIF2AK3 | GGCTGTCACTCAGGTGGC | GAGCTCCCAAGAAGGCAAGG |
| ERN1 | TAGTCAGTTCTGCGTCCGCT | TTCCAAAAATCCCGAGGCCG |
| ACTIN | AGAACTGGCCCTTCTTGGAGG | GTTTTTATGTTCCTCTATGGG |
